# Characteristics and natural history of autonomic involvement in hereditary ATTR amyloidosis: a systematic review

**DOI:** 10.1007/s10286-019-00630-y

**Published:** 2019-08-31

**Authors:** Alejandra Gonzalez-Duarte, Sergio I. Valdés-Ferrer, Carlos Cantú-Brito

**Affiliations:** Department of Neurology, Instituto Nacional de Ciencias Médicas y Nutrición Salvador Zubirán, Vasco de Quiroga 15, Co. Belisario Domínguez, Sección XVI, Tlalpan, Mexico City, 14080 Mexico

**Keywords:** ATTR amyloidosis, Dysautonomia, Autonomic dysfunction, Autonomic symptoms, Orthostatic hypotension, Familial amyloid polyneuropathy

## Abstract

**Background:**

Autonomic dysfunction is a hallmark feature of hereditary ATTR amyloidosis. The aim of this study was to summarize the characteristics and natural history of autonomic dysfunction in patients with hereditary ATTR amyloidosis.

**Methods:**

A systematic review of the natural history and clinical trials of patients with ATTR amyloidosis was performed. Alternative surrogate markers of autonomic function were analyzed to understand the prevalence and outcome of autonomic dysfunction.

**Results:**

Patients with early-onset disease displayed autonomic dysfunction more distinctively than those with late-onset disease. The nutritional status and some autonomic items in the quality-of-life questionnaires were used to assess the indirect progression of autonomic dysfunction in most studies. Gastrointestinal symptoms and orthostatic hypotension were resent earlier than urogenital complications. Once symptoms were present, their evolution was equivalent to the progression of the motor and sensory neuropathy impairment.

**Conclusion:**

The development of autonomic dysfunction impacts morbidity, disease progression, and mortality in patients with hereditary ATTR amyloidosis.

## Introduction

Hereditary ATTR amyloidosis is an autosomal dominant hereditary multisystemic disorder caused by the extracellular accumulation of abnormally misfolded transthyretin (TTR). The TTR gene provides information for the production of transthyretin, a protein that transports vitamin A (retinol) and thyroxine throughout the body. Transthyretin is produced mainly in the liver, although a small amount is also produced in the choroid plexus and in the eyes. The disease is characterized by tissue deposition of insoluble proteins and fibril aggregates oriented in a ß-pleated sheet structure that form unbranched amyloid fibrils [1]. Usual manifestations include sensory–motor neuropathy, autonomic dysfunction, cardiac, ocular, and other less common manifestations such as renal involvement. Without treatment, progressive incapacitating neuropathy, cardiac insufficiency, and cachexia due to gastrointestinal (GI) dysautonomia is common after a few years, and patients are bedridden and therefore prone to developing bedsores, venous thrombosis, muscle atrophy by denervation, infection, and ultimately death [1, 2].

Autonomic symptoms are key components of hereditary ATTR amyloidosis, are present in almost all mutations, and emerge at onset or very early afterwards; they contribute strongly to the burden of the disease [1–4]. However, these symptoms are often overlooked, as they can be hidden or interposed by more pronounced cardiovascular or GI manifestations (please refer to sections Clinical vignettes 1 and 2). Clinically, the progression of hereditary ATTR amyloidosis to late-stage disease results in patients becoming wheelchair-dependent or bedridden, either due to severe motor impairment or end-stage cardiomyopathy. Impaired mobilization further compromises the assessment of autonomic function. Therefore, autonomic manifestations are often underdiagnosed and underreported in the latter stages of the disease, and patients usually do not undergo autonomic testing.

Similar to peripheral neuropathy, the progression of autonomic symptoms is unrelenting and closely related to the progression of somatic neuropathy. However, these symptoms can worsen non-neurological manifestations such as dizziness, fatigue, or pain. Certainly, these symptoms play a critical role in the outcome, particularly in terms of cardiovascular events and sudden death [2, 3]. Signs and symptoms of autonomic disease can be assessed from diagnosis either with questionnaires or validated tests, both of which can provide objective predictors of disease progression. Surprisingly, these signs and symptoms have seldom been considered as primary endpoints in most studies assessing the natural history of hereditary ATTR amyloidosis or in clinical trials of this disorder. Thus, its natural history is poorly described.

We have conducted a systematically review of the literature with the aim to clarify the natural history and clinical characteristics of the autonomic symptoms of patients with hereditary ATTR amyloidosis. Alternative surrogate markers can be used as a reflection of the evolution of autonomic manifestations and will also be discussed in the text of this article.

## Clinical vignette 1

A 54-year-old male diagnosed with ATTR amyloidosis 3 years previously, presented with a history of numbness and burning pain in feet and hands, treated with pregabalin, and constipation, treated with stool-softeners. He also had orthostatic hypotension (OH), with a mean systolic blood pressure fall of 46 mmHg after standing for 5 min, despite treatment with fludrocortisone 0.1 mg/day. He had made an appointment for consultation due to difficulties at maintaining erections during sexual intercourse and also for advice on the use of sildenafil.

Autonomic symptoms usually occur early in the natural history of ATTR amyloidosis and can be useful criteria for calculating disease onset. The association of OH and erectile dysfunction (ED) is common in ATTR amyloidosis, particularly in patients presenting symptoms before 50 years of age. The treatment of ED is challenging in these patients because it may unmask or exacerbate drops in blood pressure. Oral phosphodiesterase inhibitors, such as sildenafil citrate or tadalafil, are efficacious in the treatment of ED, but are associated with major risks in the presence of OH [5]. Consequently, lying and standing blood pressure should be measured in any patient wishing to start on therapy with an oral phosphodiesterase inhibitor. If possible, a clinical trial should be performed at the medical doctor’s office to determine if the patient is a candidate for this type of treatment. Additional recommendations are to avoid other activities or drugs that may trigger hypotension, such as ingestion of alcohol, use of tricyclic antidepressants, diuretics, or alpha-1 blockers, and avoidance or delay of parasympathetic maneuvers, such as straining while urinating, having a bowel movement, or coughing vigorously. In most cases, other non-pharmacological treatments for ED are safer to use, such as vacuum constriction devices, intracavernosal and intraurethral devices, with or without prostaglandin E1 (PGE1).

## Clinical vignette 2

A 45-year-old man with a TTR Ser50Ala mutation arrived at the emergency room after experiencing syncope after running 1 mile. His most recent echocardiogram showed moderate myocadiac amyloid infiltration, with a fraction ejection of 56%. An electrocardiogram showed complete atrioventricular conduction block and persistent sinus bradycardia with pauses that required cardiac pacing. Following the procedure, the systolic blood pressure of the patient continued to drop > 50 mmHg after standing. Further treatment was deferred until consultation with a neurologist.

OH resulting from autonomic dysfunction is a common cause of syncope in patients with cardiac amyloidosis [6]. Syncope is mainly associated with arterial hypotension. Heart failure prevents the heart from pumping efficiently or rapidly enough to compensate for the drop in blood pressure that occurs upon standing. Thus, vasodilation with an inability to increase cardiac output due to poor contractility reserve results in exertional syncope. Less frequently, ventricular and atrial arrhythmias with rapid ventricular rates can also result in syncope. Ambulatory cardiac and blood pressure monitoring can be helpful to assess each of these conditions. Early pacemaker or implantable cardioverter–defibrillator placement should be considered, as individuals with these conditions are likely to experience sudden death. Digoxin has been associated with higher toxicity and it is proscribed. The efficacy of angiotensin converting enzyme inhibitors, angiotensin receptor blockers, and beta-blockers is still undefined in amyloidosis [7]. Low-dose diuretics improve the symptoms of heart failure but tend to aggravate hypotensive symptoms; if their use is judged necessary, low doses, preferably taken at night, are recommended. Midodrine, an alpha-1 adrenergic stimulant, is often necessary in high doses to maintain blood pressure. The supine hypertension that is common with midodrine use is not seen in individuals with cardiac amyloidosis, so these patients can use large doses without concern [8], and its use as a prophylactic before exercise may be helpful [8].

## Methods

**Fig. 1:**
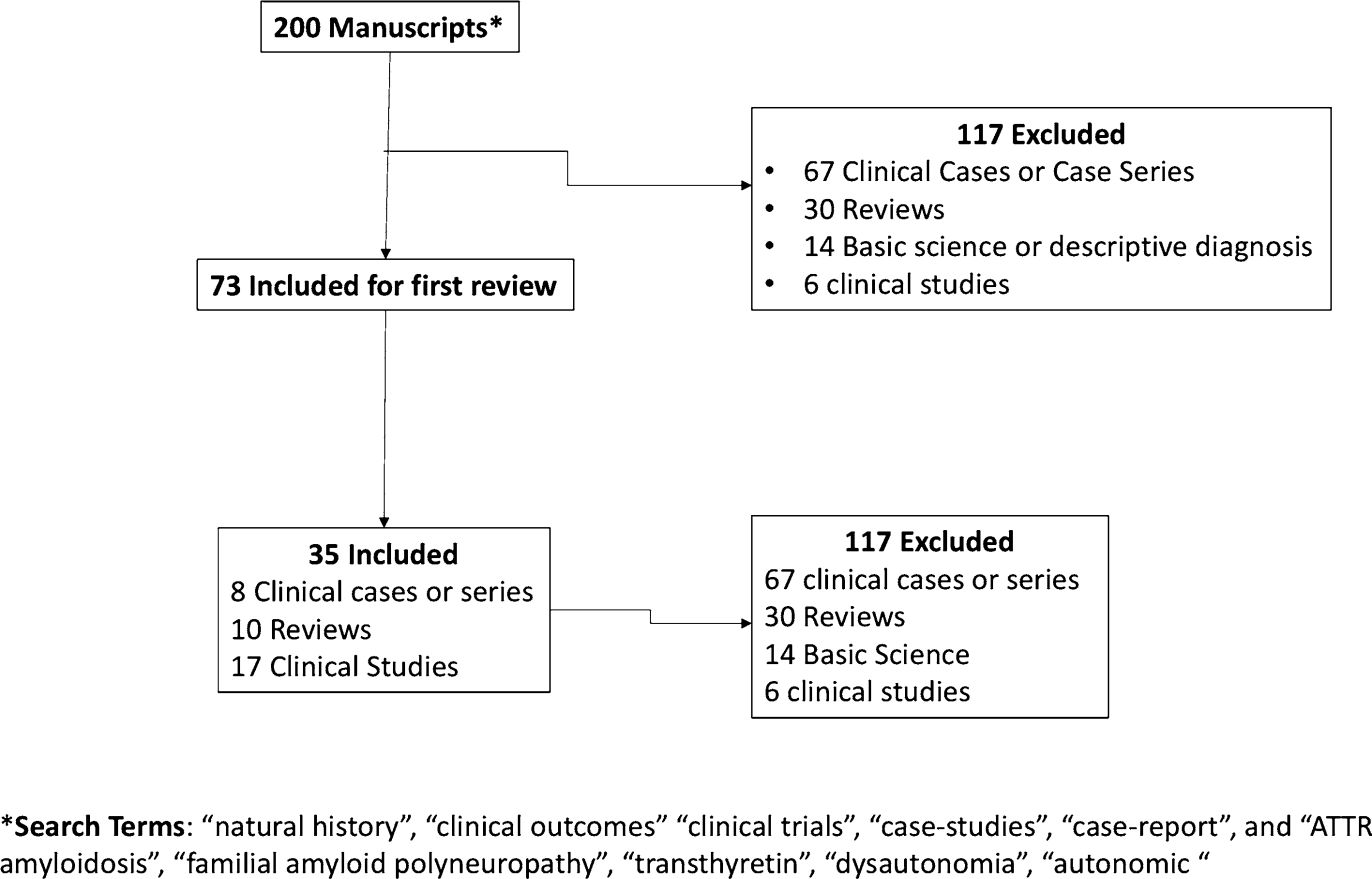
PRISMA [9] flow chart summarizing the selection process of articles included in this systematic review

This sytematic review was conducted according to the PRISMA statement for reporting systematic reviews [9]. A literature search was performed using the terms “natural history,” “clinical outcomes,” “clinical trials,” “case studies,” and “case report,” and “ATTR amyloidosis,” “familial amyloid polyneuropathy,” “transthyretin,” “dysautonomia,” and “autonomic.” Most of the articles identifed with a combination of these terms were clinical series of cases describing specific mutations. Of these, only the ones with a complete clinical description of the autonomic symptoms were included in the review. The remaining articles identified were reviews, clinical studies, and clinical trials. All articles were studied, and symptoms or signs of autonomic dysfunction were registered. Of the 200 articles identified by the search terms, 35 were included in our systematic review (Fig. 1 and Table 1). Six abstracts with relevant autonomic information were included.

**Table 1:** Clinical studies assessing autonomic symptoms in hereditary ATTR amyloidosis

Title	Year	Patients and stage disease	Objectives	Autonomic findings
Corino–Andrade disease (familial amyloidotic polineuropathy type I) in Spain: urological and andrological disorder [10]	1997	12 patients	To describe urological and sexual disorders	Principal findings were low bladder pressure and bladder neck obstruction with micturition disorders. Males also showed impotence and retrograde ejaculation
Neurophysiological evaluation of sexual dysfunction in familial amyloidotic polyneuropathy: Portuguese type [11]	1997	15 male patients (mean disease duration 5.2 ± 2.2)	To correlate clinical and EMG scores in sacral arch functioning (PEP, BCR and SSR)	PEP and BCR had statistical significant correlation with clinical and EMG scores
Rapid intestinal transit as a primary cause of severe chronic diarrhea in patients with amyloidosis [12]	2003	3 patients (symptom duration: 4 years)	To assess the mechanisms of amyloid diarrhea through pathophysiological studies	Diarrhea caused by autonomic neuropathy, not by malabsorption, bacterial overgrowth, bile acid malabsorption, or epithelial cell malabsorption of electrolytes
Autonomic dysfunction in FAP: its therapeutic effect by liver transplantation [13]	2006	50 patients(< 5 years from onset)	To assess liver transplantation outcomes	Early transplantation provides better chance of improving gastrointestinal autonomic symptoms
Quantitative sensation and autonomic test abnormalities in transthyretin amyloidosis polyneuropathy [14]	2009	36 patients	To assess QAT and QST	Autonomic prominent dysfunction in most patients, as frequent as large fiber disfunction. Autonomic tests rationale for assessing severity of ATTR amyloidosis
Parenteral nutrition improves nutritional status, autonomic symptoms, and QOL in transthyretin amyloid polyneuropathy [15].	2016	2 patients (late stage in treatment with tafamidis and OLT)	To evaluate parenteral nutrition	Parenteral nutrition improved nutritional status, autonomic symptoms (OH, nausea, diarrhea) and QOL
The value of electrochemical skin conductance measurement using Sudoscan® in the assessment of patients with FAP [16]	2018	126 patients(asymptomatic, pauci-symptomatic, moderate and advanced)	To reappraise the value of Sudoscan® to assess small autonomic fibers	Measurements reduced in 24% of clinically asymptomatic patients, 40% of pauci-asymptomatic patients, 65% with moderate, and 92% with advanced disease
Assessment of autonomic innervation of the foot in familial amyloid polyneuropathy [17]	2019	21 patients (clinically asymptomatic, moderate, or advanced neuropathy)	To assess sudomotor tests (Neuropad® and Sudoscan®) and associations with a QST, NCS, and LEP	Sudomotor function assessed through Neuropad® and Sudoscan® proved to be early markers of neuropathy and correlated with NIS. Sudoscan could be valuable to follow progression

*ATTR *ATTR amyloidosis,* BCR* bulbocavernous reflex,* EMG* electromyography,* FAP* familial amyloidotic polyneuropathy,* LEP* laser evoked potential,* NCS* nerve conduction study,* NIS* Neuropathy Impairment Score,* OH* orthostatic hypotension,* OLT* orthotopic liver transplant,* PEP* pudendal evoked potentials,* QAT* quantitative autonomic test,* QOL* quality of life,* QST* quantitive sensation test,* SSR* sympathetic skin response

## Results

We identified ten articles on clinical trials and clinical trial extensions that described the autonomic characteristics of the patient study population and ten review articles that focused on some aspects of autonomic function. The remaining studies included case series of different populations that were chosen for inclusion in this review because they provided an explanation of the autonomic symptoms or signs. Abstracts from relevant meetings were also included (Fig. 1; Table 1).

### Prevalence

**Fig. 2:**
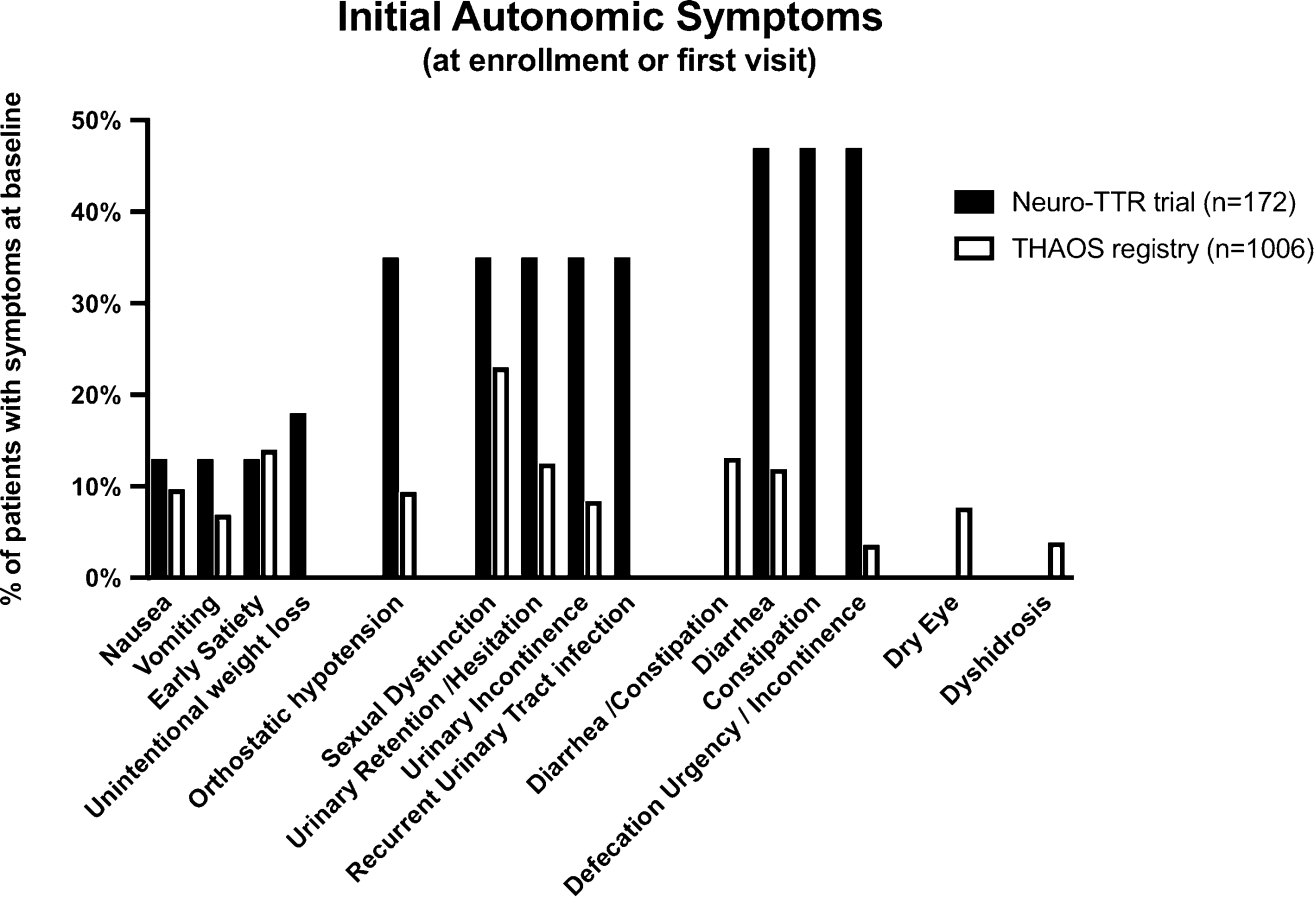
Autonomic symptoms at enrollment or first visit in patients with hereditary ATTR amyloidosis enrolled in the Neuro-TTR trial and the THAOS registry. Entry criteria for Neuro-TTR trial was disease stage I-II (patients still able to walk). The THAOS registry had no entry limitations and patients could be enrolled at any stage, including asymptomatic patients

Autonomic manifestations were present in 50–80% of the patients [18, 19]. The most common findings were OH, diarrhea, constipation, alternating diarrhea and constipation, erectile dysfunction, urinary incontinence, and xerostomia (Fig. 2) [18–23]. Different mutations carried different risks for autonomic disfunction. In the THAOS registry, prevalence of autonomic dysfunction was higher in patients with Val30Met mutations and lower in those carrying a cardiac mutation [18, 19]. Autonomic dysfunction was described as moderate in patients with the Glu89G and Phe64Leu mutations and severe in those with the Thr49Ala mutation [21]. Autonomic disturbances were not present in most patients with Phe64Leu mutations with new-onset symptoms, but these disturbances occurred in all patients within approximately 4 years following symptom onset [21]. In contrast, autonomic symptoms were the presenting symptom in 50% of patients with the Thr49Ala mutation [21]. Autonomic manifestations were also common in patients with Ser50Arg, Ser52Pro, and Gly47Ala mutations [22]. In the Neuro-TTR trial, patients with the Thr60Ala and Ser77Tyr mutations had a higher percentage of autonomic involvement [23]. Also, patients with Val30Met mutations had the shortest time from the first ATTR amyloidosis-related symptom to the first autonomic symptom, with a mean time from onset of 2.7 years [19].

### Timing of symptoms

It is difficult to estimate the exact timing of the onset of autonomic symptoms. Often, in many studies, early autonomic symptoms were overlooked and identified retrospectively, only after other non-autonomic symptoms occurred. For example, in Sicily, one patient was misdiagnosed as recurrent vasovagal syncope over a period of 4 years, whereas the patient probably had OH because somtime later he experienced paresthesias in the hands and impotence, and mixed parasympathetic and sympathetic dysfunction was detected [21].

In most studies, the emergence of autonomic symptoms depended on the type of onset of the disease. In two studies involving Japanese patients, the characteristics of early-onset cases in 81% of the patient population from endemic foci included the presence of sensory dissociation and marked autonomic dysfunction associated with a predominant loss of small-diameter and unmyelinated nerve fibers [24, 25]. These characteristics were not common in the late-onset cases from non-endemic areas, where autonomic dysfunction was less prevalent (around 60%) and of milder intensity [4]. In the THAOS Registry consisting of a population of 781 symptomatic patients, autonomic neuropathies were associated more frequently with an early onset of disease than with late-onset disease [18]. Patients with autonomic dysfunction at enrollment in the THAOS Registry were younger and had longer duration of ATTR amyloidosis symptoms when compared with those without autonomic dysfunction at enrollment [19]. Of these former patients, 35% experienced an autonomic symptom as their first ATTR amyloidosis manifestation [19].

Also, different autonomic manifestations may occur at different times. In studies involving patients with early-onset disease, GI dysfunction and OH emerged in the early phase of the disease, whereas urinary manifestations appeared halfway through the course of the disease [4]. A study from Japan that included early-onset cases from an endemic foci of patients with Val30Met mutations reported that the GI symptoms constituted the initial symptom in nearly half of the affected individuals, but in late-onset patients, diarrhea and constipation did not precede symptoms of somatic neuropathy, although they tended to appear in the early phase of the disease [24]. In a study conducted in Mexico, autonomic symptoms were the initial presentation in 4% of individuals with the Ser50Ala, Ser52Pro, and Gly47Arg mutations [22]. In this population, patients with Coutinho’s stage (Table 2) 0-I showed alterations in different autonomic tests, such as drops in systolic blood pressure during the tilt-test and abnormalities in the E:I ratio and the Valsalva ratio [22]. Likewise, 70% of the population in the Neuro-TTR trial had all four symptomatic categories: muscle weakness, sensory manifestations, neuropathic pain, and autonomic symptoms, whereas only 6% did not have autonomic symptoms at onset [23]. In this patient population, over 50% of the patients had a history of GI manifestations, such as recurring diarrhea, constipation, defecation urgency, GI mobility disorder, or GI hypomotility [23].

**Table 2:** Coutinho staging [26]

Stage	Description
Stage I	Unimpaired ambulation; mostly mild sensory, motor, and autonomic neuropathy in the lower limbs
Stage II	Assistance with ambulation required; mostly moderate impairment progression to the lower limbs, upper limbs, and trunk
Stage III	Wheelchair-dependent or bedridden; severe sensory, motor, and autonomic involvement of all limbs

### Evaluation of symptoms

The most common primary outcome of clinical trials on ATTR amyloidosis is neurological progression [27–31]. The Neuropathy Impairment Score (NIS) (Table 3) and its variations are the most popular tools to assess such progression. NIS+7 is a widely used outcome [27, 28]. The developers of this measure performed a retrospective analysis of 97 untreated patients with familial amyloid polyneuropathy and proposed several modifications to improve sensitivity: to use only the amplitudes of the compound muscle action potentials and sensory nerve action potential; to add somatotopic quantitative sensory testing; and to perform new autonomic measures (e.g., quantitative sudomotor testing or heart rate variability measures) [32]. The incorporation of the modified instrument helped determine contributing autonomic function. Heart rate variability is a reliable marker of autonomic alterations and has shown to be impaired very early in the disease. Unfortunately, it may not be feasible to obtain this measure in some patients due to arrhythmias [33].

**Table 3:** Polyneuropathy Disability Scale staging [26]

Polyneuropathy Disability Scale	Description
Stage 0	No impairment
Stage I	Sensory disturbances but preserved walking capability
Stage II	Impaired walking capability but ability to walk without a stick or crutches
Stage IIIA	Walking only with the help of one stick or crutch
Stage IIIB	Walking with the help of two sticks or crutches
Stage IV	Wheelchair-dependent or bedridden

### Progression of autonomic dysfunction

Autonomic symptoms parallel the progression of sensory and motor dysfunction. In the Neuro-TTR trial, autonomic symptoms were progressive and corresponded to the neurological impairment, as assessed using the Polyneuropathy Disability Scale (PND) staging (Table 4), with 50% of patients with PND I having autonomic manifestations, 70% of the patients in PND II, and up to 90% of patients in PND III/IV [23]. The same was observed with the autonomic sudomotor innervation of the lower extremities, with the patients with advanced disease showing more disease progression [17, 16]. Patients with ATTR amyloidosis with polyneuropathy in trials of tafamidis, patisiran, and inotersen showed improvements in nutritional status and autonomic function, as assessed based on the items of diarrhea, vomiting, and dizziness of the Norfolk Quality of Life-Diabetic Neuropathy instrument (Norfolk QoL-DN) [29–31]. The APOLLO study evaluated autonomic symptoms with the Compass-31 scale, reporting great improvement in patients using patisiran at 18 months when compared to placebo [30]. At 18 months, orthostatic tolerance and GI domains showed the greatest improvement [34, 35].

**Table 4:** The Neuropathy Impairment Score and its iterations

NIS and its iterations	Complete name	Description
NIS	Neuropathic Impairment Score	Neuropathy impairment exam that evaluates motor function, reflex abnormalities and sensory loss
NIS-LL	NIS in the Lower Limbs	Limited to the lower limbs
NIS+7	NIS+7	Adds nerve conduction studies
mNIS+7	Modified NIS+7	Compounds smaller fiber function (vibration or touch-heat-pain sensibility) and autonomic testing (heart rate decrease with deep breathing or OH measurements).

### Quality of life and autonomic relationship

Most clinical trials have used the Norfolk QoL-DN scale, which evaluates some autonomic symptoms, to evaluate the quality of life. This instrument showed that worsening of the quality of life is closely related to the progression of neuropathy, asevaluated with mNIS+7. When compared to placebo, tafamidis, inotersen, and patisiran have demonstrated improvement across all domains of the Norfolk QOL-DN, including the autonomic domains [29–31].

## Discussion

**Fig. 3:**
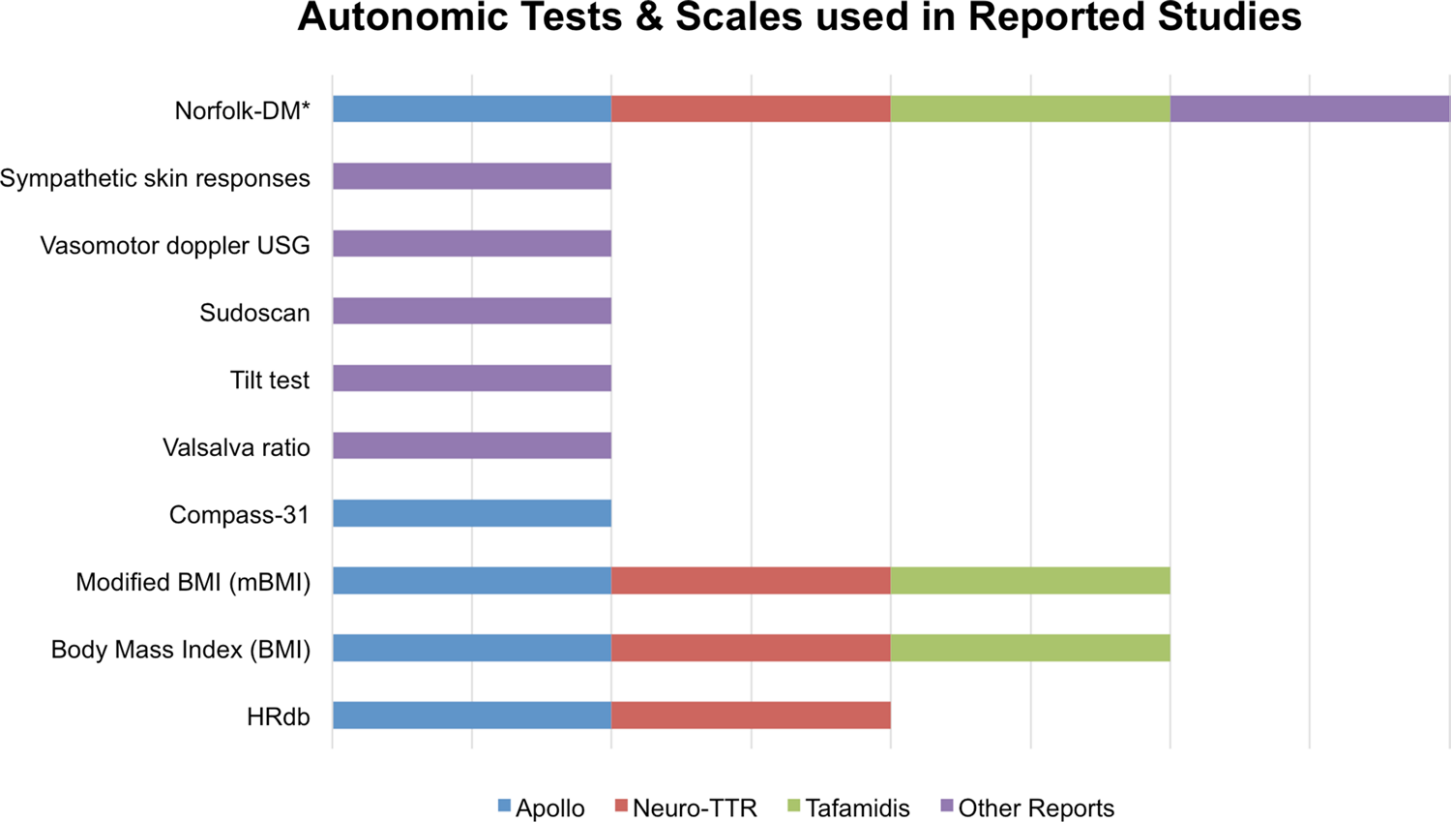
Common autonomic assessments and tests used in hereditary ATTR amyloidosis clinical studies. The Norfolk DM questionnaire assesses the following autonomic items: dizziness, vomiting, and diarrhea.* Norfolk QoL-DN* Norfolk Quality of Life-Diabetic Neuropathy instrument,* HRdb* heart rate decrease with deep breathing

As shown by the clinical vignettes, the clinical manifestations of autonomic dysfunction in patients with hereditary ATTR amyloidosis may be masked by other non-autonomic symptoms, and they can also limit the therapeutic options. The prevalence and natural history of autonomic dysfunction has not been fully elucidated, primarily because it is a very rare disease and most of the published reports are cases or case studies. However, prevalence seems to be more common in patients with certain types of mutations, in younger patients, and in those with early-onset disease. Symptom progression often parallels peripheral neuropathy progression. The autonomic components of the Norfolk QOL-DM questionnaire and assessment of nutritional status are the most common endpoints used in clinical trials to assess autonomic function, although the COMPASS-31 questionnaire was used in one trial and showed good results (Fig. 3). More complex measurements are challenging due to the multisystemic involvement of the disease. For example, heart rate variability is limited in patients with arrhythmias, and the assessment of orthostatic hypotension is difficult in patients who are unable to stand up due to severe motor neuropathy.

The limitations of this review include the poor characterization of the progression of the autonomic symptoms and the absence of robust, objective indicators of the progression of the disease.

## Conclusions

Autonomic dysfunction adds complexity to the burden of hereditary ATTR amyloidosis. Assessing autonomic symptoms carefully will help the clinician to better understand the multisystemic complaints of patients with hereditary ATTR amyloidosis and will facilitate the treatment of certain conditions. Better biomarkers to assess the onset and progression of the autonomic dysfunction are needed.
